# Not Enough Homœopathy Taught

**Published:** 1897-09

**Authors:** J. M. Selfridge

**Affiliations:** Oakland, Cal.


### NOT ENOUGH HOMŒOPATHY TAUGHT.

J. M. Selfridge, M. D., Oakland, Cal.

In talking with a Medico one day, 'twas said to me,
I much regret to say it, but the same it is too true,
That in our college Medics the professors, if they knew,
Teach not unto the students, except in small degree,
The science therapeutics known as Homœopathy.

That this was true, in my reply, I said, I knew before,
That fairness did not fill the minds of all of those who taught
When teaching trusting students such therapeutic rot,
In which was nothing new at all, but much from days of yore,
And nearly everything they taught was allopathic lore.

Besides, it is not honest, when they go expecting more
Of the law of true *Similia*, the only law of cure;
When *Similia Similibus* is the *Curanter* sure,
To teach them how to palliate, and all such stuff, before
They're made to understand true homœopathic lore.

Protest we may, they still pursue their methods just the
same.
They yearly send young Medicos, in name of science true,
Who, seeking out a clientele, they all (except a few),
A mongrel sort of practice give, and seem to think it's game,
When told what's true, that all they do, is homœopathic but
in name.

It's fraud upon the public all, in that it does deceive,
When asked for true *Similia*, to give crude drugs the most,
It gives unto the allopath a chance for us to roast,
They say we all are practicing what *none* of us believe,
And, doubtless, think they honest are—they say we *all* de-
ceive.

Enough of this, let's turn the page and see what we can do.
A club we'll have, we'll study hard, *The Organon* we'll teach.
For those who wish to know the truth we'll surely outward
reach.
We'll ground them well in what is old, as well as what is new,
And do the very best we can to teach at least a few.

Unto what class of Medicos shall we our call extend?
To males alone? To females? Yes; for they are doctors,
too.
Or to such men whom we all know in doctrine to be true?
To men alone say two or three, while others, they contend
That all such thoughts are narrow ones—to *broadness* is the
trend.

And this, I think, is just the thing—the women, they are
here;
They're surely in as Medicos, we cannot keep them out;
And if we try we'll surely fail—they know what they're
about.
Then teach them well the doctrine pure, for though I'm not
a seer,
They're here to stay, say what we may; then teach and have
no fear.

There's one objection, I have heard, 'gainst women doctors
urged.
The code of ethics they don't heed, but kick the traces o'er.
They seem to think their crinoline protects them evermore
In everything they do, or say, e'en far beyond the verge
Of ethics' laws, that's due to all, though pantaloon'd or
serged.

There's one thing sure, with all our pride, it may as well be
said,
The world's not ours, 'tis long and wide, and ev'ry one may
choose

A work in life, 'tis ope to all, be't medicine or muse;
For men and girls do what they please (providing they don't
wed);
Then don't forget, we men don't own a corner lot in Med.

